# Novel Histomorphometrical Approach to Evaluate the Integration Pattern and Functionality of Barrier Membranes

**DOI:** 10.3390/dj9110127

**Published:** 2021-10-25

**Authors:** Nicola Ottenbacher, Said Alkildani, Tadas Korzinskas, Jens Pissarek, Christian Ulm, Ole Jung, Bernd Sundag, Olaf Bellmann, Sanja Stojanovic, Stevo Najman, Werner Zechner, Mike Barbeck

**Affiliations:** 1 Clinical Division of Oral Surgery, Dental University Clinic, Medical University of Vienna, 1090 Vienna, Austria; ottenbacher.nici@gmail.com (N.O.); christian.ulm@tuvienna.os (C.U.); werner.zechner@meduniwien.ac.at (W.Z.); 2 BerlinAnalytix GmbH, 12109 Berlin, Germany; said.alkildani@berlinanalytix.com (S.A.); tadaskorzinskas@yahoo.de (T.K.); 3 Biotrics Bioimplants AG, 12109 Berlin, Germany; jens.pissarek@biotrics.com; 4 Clinic and Policlinic for Dermatology and Venereology, University Medical Center Rostock, 18057 Rostock, Germany; ole.tiberius.jung@gmail.com (O.J.); b.sundag@gfn-selco.de (B.S.); 5 Research Institute for Farm Animal Biology (FBN), 18196 Dummerstorf, Germany; bellmann@fbn-dummerstorf.de; 6 Scientific Research Center for Biomedicine, Department for Cell and Tissue Engineering, Faculty of Medicine, University of of Niš, 18000 Niš, Serbia; sanja.genetika.nis@gmail.com (S.S.); stevo.najman@gmail.com (S.N.); 7 Department of Biology and Human Genetics, Faculty of Medicine, University of Niš, 18000 Niš, Serbia; 8 Austrian Cluster for Tissue Regeneration, 1200 Vienna, Austria; 9 Department of Ceramic Materials, Chair of Advanced Ceramic Materials, Institute for Materials Science and Technologies, Technical University Berlin, 10623 Berlin, Germany

**Keywords:** collagen, hyaluronic acid, barrier membranes, guided bone regeneration (GBR), cellular immigration, biomaterial integration, histomorphometrical analysis

## Abstract

GBR (guided bone regeneration) is a standard procedure for building up bony defects in the jaw. In this procedure, resorbable membranes made of bovine and porcine collagen are increasingly being used, which, in addition to many possible advantages, could have the potential disadvantage of a shorter barrier functionality, especially when augmenting large-volume defects. Thus, it is of importance to evaluate the integration behavior and especially the standing time of barrier membranes using specialized methods to predict its respective biocompatibility. This study is intended to establish a new histomorphometrical analysis method to quantify the integration rate of collagen-based barrier membranes. Three commercially available barrier membranes, i.e., non-crosslinked membranes (BioGide^®^ and Jason^®^ membrane), a ribose-crosslinked membrane (Ossix^®^ Plus), and a newly developed collagen–hyaluronic acid-based (Coll-HA) barrier membrane were implanted in the subcutaneous tissue of 48 6–8-week-old Wistar rats. The explants, after three timepoints (10, 30, and 60 days), were processed and prepared into histological sections for histopathological (host tissue response) and histomorphometrical (cellular invasion) analyses. 10 days after implantation, fragmentation was not evident in any of the study groups. The sections of the Coll-HA, Jason^®^ and BioGide^®^ membranes showed a similar mild inflammatory reaction within the surrounding tissue and an initial superficial cell immigration. Only in the Ossix^®^ Plus group very little inflammation and no cell invasion was detected. While the results of the three commercially available membranes remained intact in the further course of the study, only fragments of the Coll-HA membrane were found 30 and 60 days after implantation. Histomorphometrically, it can be described that although initially (at 10 days post-implantation) similar results were found in all study groups, after 30 days post-implantation the cellular penetration depth of the hyaluronic acid-collagen membrane was significantly increased with time (**** *p* < 0.0001). Similarly, the percentage of cellular invasion per membrane thickness was also significantly higher in the Coll-HA group at all timepoints, compared to the other membranes (**** *p* < 0.0001). Altogether, these results show that the histomorphometrical analysis of the cellular migration can act as an indicator of integration and duration of barrier functionality. Via this approach, it was possible to semi-quantify the different levels of cellular penetration of GBR membranes that were only qualitatively analyzed through histopathological approaches before. Additionally, the results of the histopathological and histomorphometrical analyses revealed that hyaluronic acid addition to collagen does not lead to a prolonged standing time, but an increased integration of a collagen-based biomaterial. Therefore, it can only partially be used in the dental field for indications that require fast resorbed membranes and a fast cell or tissue influx such as periodontal regeneration processes.

## 1. Introduction

Guided bone regeneration (GBR) was established in the 1980s and is nowadays seen as a standard therapeutic procedure for regenerate bone defects in implantology as well as in oral and maxillofacial surgery [1]. Current systematic reviews show that the GBR technique is a reliable method for alveolar ridge preservation/augmentation [2]. By using absorbable or non-absorbable barrier membranes, the bone defect area is separated from the oral flora [3]. Technically, the membrane forms a barrier between the soft tissue and the bone defect area and thus prevents the non-osteogenic cell population from migrating into the area of the bony defect and offering the osteogenic cell population of the original bone the opportunity to grow [4]. Beside this cellular occlusion property, the barrier membrane should provide space maintenance, preventing the soft tissue from falling into the defect area which can lead to increased inflammation and ultimately, implantation failure [5]. Alongside these traditional properties, special properties of the barrier membranes are necessary and currently being intensively researched [6,7]: (i) biocompatibility: is responsible for cell attachment and tissue integration, reduces or prevents inflammatory degradation processes, (ii) handling: safe clinical applicability and easy intraoperative handling, (iii) wound healing: stabilization of blood clot, and (iv) tissue integration: transmembrane angiogenesis (enables early ingrowth of blood vessels).

During the initial phase of the GBR technique, non-resorbable membranes were predominantly used. However, resorbable membranes are increasingly common in clinical use today [8]. The advantages of resorbable membranes are that no second surgical intervention is necessary to remove the membrane and the fact that its self-resorbing properties can lower the chances of wound dehiscence. In addition, these membranes are preferable due to their better price-performance ratio and reduced patient morbidity [9].

The resorbable membranes can be divided into two categories: synthetic and natural membranes. Natural membranes are mainly based on collagen of animal origin, silk fibroin, or chitosan [10]. Synthetic polymer-based membranes are mainly produced from blends of aliphatic polyesters [11].

The fact that it has a structural component and regenerative properties due to its functionality as an extracellular matrix protein, speaks for the use of collagen [12]. Collagen as a biomaterial is mostly bovine or porcine-derived and characterized as collagen type I and III. The most common extraction sites are dermis, pericardium, and Achilles tendons [13]. Collagen has a chemotactic effect on fibroblasts (tissue integration) and promotes the formation of new blood vessels [14]. Collagen barriers have already been extensively investigated in animal experiments and also in clinical studies [15,16,17,18,19,20]. Thereby, especially the clinical results are comparable with those of non-resorbable membranes [21]. In this comparison, collagen membranes also show a lower incidence of spontaneous exposures [11]. In addition, collagen-based biomaterials have increased resistance to infection compared with permanent implants [22]. This can be due to (i) the shorter service life of collagen-based implants as infection can be early or latened and, (ii) increased vascularization of collagen-based implants, which in turn increases the influx of antibacterial molecules and immune cells [23].

A potential disadvantage of native collagen, however, is its relatively short barrier functionality, as it is rapidly broken down by tissue-specific proteases, collagenases and cell types like macrophages [20,24]. For this reason, the demands on the barrier functionality and dimensional stability are increased, especially when augmenting large-volume defects, where a longer regeneration time can be assumed [3].

Hyaluronic acid (HA) is a non-sulfated glycosaminoglycan being a component of the extracellular matrix in the different tissues [25]. It supports the viscoelasticity of the tissue, acts as a hydrating component, and is a ligand for CD44 guiding cellular motility and adhesion [25,26,27]. Therefore, HA has been investigated for its wound-healing capacity, as well as its regenerative capacities in the context of maxillofacial and orthopedic surgeries triggering processes like osteoblast or endothelial cell migration [26,28,29,30]. Therefore, a novel barrier membrane membrane for GBR applications was developed on basis of native dermal collagen via complexing with HMWHA in order to trigger tissue integration and wound-healing.

The resistance of the collagen fibrils to decomposition correlates directly with the density of the intermolecular crosslinks, hindering the access of the hydrolytic water molecule [31]. Therefore, to oppose the disadvantage of a quicker resorption and a shorter barrier functionality, various methods have been developed to crosslink collagen, i.e., aldehyde fixatives, imides, and treatments such as hydration and radiation [32]. The major drawbacks of such treatments, however, are resulting potential bioincompatibilities correlated with difficult-to-control degrees of crosslinking, which could lead to premature breakdown of the membrane, contrary to the intentions of increasing the standing time [33,34]. Thus, it is of great interest to analyze the integration behavior even in view of the barrier functionality of such biomaterials—especially in case of new material development to predict the standing time of barrier membranes and simultaneously its biocompatibility.

As a current standard, cellular infiltration (as a function of barrier functionality) of barrier membranes is only qualitatively analyzed through histopathological methods [6,33,34]. This analysis is done through the identification of the different cells of the immune system, their location at the surface/within the biomaterial, presence of multinucleated giant cells (MNGCs), and the occurrence of fibrosis/neovascularization/necrosis [18]. However, no quantitative approach to analyze the integration behavior of collagen-based barrier membranes has been developed until now. Thus, the present study was conducted aiming to investigate a quantitative method of evaluating the cellular migration into barrier membranes, as even this is essential in understanding and predicting the biofunctionality of resorbable barrier membranes.

## 2. Materials and Methods

### 2.1. Barrier Membranes

Four different barrier membranes, which are described below, were used for the histomorphometrical analysis of this study.

#### 2.1.1. Test Membrane

The newly developed Coll-HA barrier membrane was produced by non-covalently complexing a porcine dermis-derived collagen membrane with hyaluronic acid. For preparation of the membrane, the collagen membrane was combined with 50 mg of hyaluronate with a molecular weight of 2 MDa in wet condition and lyophilization was conducted. Afterwards, the membrane was sterilized by ethylene oxide (EO) and packed.

#### 2.1.2. Commercially Available Membranes (Control Membranes)

The Jason^®^ membrane (botiss biomaterials GmbH, Zossen, Germany) is based on native collagen won from porcine pericardium. The standardized production process includes a controlled selection of the donor animal through veterinary controls. Further steps include cleaning, including wet chemical processes and lyophilization, as well as a final sterilization step using EO gas (*Jason Membrane*; *Botiss Biomaterials*, 2020).

The Ossix^®^ Plus membrane (Datum Dental, Lod, Israel) is a ribose cross-linked collagen membrane and contains porcine collagen type I, obtained from the Achilles tendon of cattle. It is described that the membrane is initially cleaned by several “processing steps” and returned to its monomeric stage by enzymatic treatment with pepsin in order to enable a more efficient removal of potentially immunogenic telopeptides in native collagen. In addition, the cross-linking of the collagen is prepared with the help of ribose with the so-called GLYMATRIX^®^ technology (*Regedent—Ossix Plus Broschüre*, 2020).

In case of the Bio-Gide^®^ membrane (Geistlich Pharma AG, Wolhusen, Switzerland), it is stated that the precursor tissue from porcine dermis is purified by a multi-stage process that allows to remove fat and other tissue components. As a final step, the BioGide^®^ membrane is sterilized via gamma irradiation (*Geistlich BioGide^®^*, 2020).

### 2.2. In Vivo Study Design, Im- and Ex-Plantation

The in vivo study was initially authorized by the local Ethical Committee of the Faculty of Medicine (University of Nilš, Niš, Serbia) based on the decision number 323-07-00073/2017-05/7 of the Veterinary Directorate of the Ministry of Agriculture, Forestry and Water Management of the Republic of Serbia. The in vivo study was also performed at the Faculty of Medicine of the University of Niš including as well as the animal housing. All animals were kept under standard conditions, such as artificial light, water ad libitum, and regular rat pellet. Standard pre- and post-operative care was ensured.

The biomaterials were obtained from a total of 48 female, 6–8-week-old Wistar rats from the Military Medical Academy (Belgrade, Serbia) and randomly divided into four study groups. Each of the study groups contained 12 test animals, with 4 of the animals being used for the implantation of the respective biomaterial per time point (n = 4), 10, 30 and 60 days. The subcutaneous implantation was carried out according to a fixed protocol described by Barbeck et al. [18]. Briefly, the animals were sedated by intraperitoneal injection (10 mL ketamine (50 mg/mL) with 1.6 mL xylazine (2%)), shaved and disinfected before an incision was made on the rostral part of the interscapular region and the biomaterials were implanted into the subcutaneous pocket was performed. The wounds were then sutured.

The implantation area was explanted together with the peri-implant tissue after 10, 30 or 60 days after implantation, depending on the grouping. For this purpose, the animals were euthanized using Euthasol (400 mg/mL). The explanted tissue was fixed in a 4% formalin solution for 48 h and then stored in phosphate-buffered saline until it could be processed histologically.

### 2.3. Histological Workup and Staining

For histological workup, the tissue explants were initially cut into two segments of identical dimensions and dehydrated using a series of increasing alcohol concentrations. After a xylene exposure, paraffin embedding was performed. Sections were prepared with a thickness of 3–5 μm by means of a rotation microtome (SLEE, Mainz, Germany). Two slides of every tissue explant were used for histochemical staining, i.e., hematoxylin/Eosin (H&E).

### 2.4. Histopathological and Histomorphometrical Analysis

After the histological workup, histopathological and histomorphometrical analyses were conducted. In case of the histopathological analysis, the H&E slides were observed qualitatively, and the evaluation included the following parameters in the context of the early and late tissue reaction related to the implants: fibrosis, bleeding, necrosis, vascularization and the presence of neutrophils, lymphocytes, plasma cells, macrophages and multinucleated giant cells (MNGCs). This qualitative analysis was performed by means of a light microscope (Axio Scope. A1) and an Axiocam 305 color digital camera in combination with ZEN Core software (all: Zeiss, Oberkochen, Germany).

The newly developed histomorphometrical analysis method included the initial digitization of the tissue slides stained with Masson’s trichrome by means of a scanning microscope setting, i.e., the Axio Scope. A1 microscope, the Axiocam 305 color digital camera and an automatic scanner table (Maerzhaeuser, Wetzlar, Germany) and the ZEN Core software (all from Zeiss, Oberkochen, Germany).

**Figure 1:**
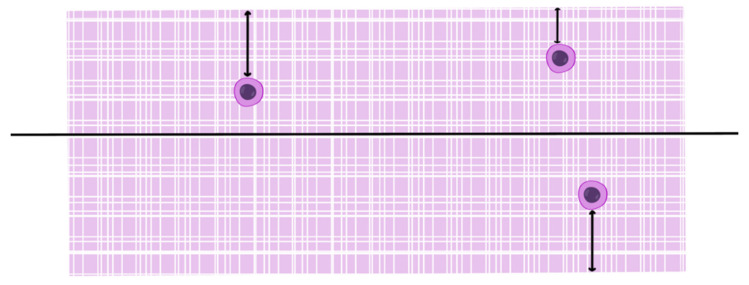
Schematic illustration of the histomorphometrical analysis of the cellular penetration depth measurements of the membranes. The membranes (checkered area) were divided into two approximately identical examination areas (black line) to measure the respective migration of cells from both material surfaces (double arrows) for calculation of the percentage migration depth of the cells.

Then, the respective area of the membrane was divided into two approximately identical examination areas, each representing one half of the membrane (Figure 1). Initially, the thickness of the two halves of membranes were calculated by measuring the respective thickness as previously described [6,18]. The next step of the histomorphometrical analysis included the manual measurement of the migration depth of the cells into the membrane, starting on both outer surfaces, via the software ImageJ (Figure 1).

Based on these measurement data, the mean value of the immigration depth (µm) and the percentage migration depth of the cells (%) were calculated. The percent depth of immigration was then calculated by relating the cellular immigration to the average width of the membrane part in question at the respective timepoint. For this, the width of the membrane in the relevant membrane area was measured 10 times and then averaged.

### 2.5. Statistical Analysis

For the statistical analysis, quantitative data after an analysis of variance (ANOVA) were presented as the mean ± standard deviation, which enables the data from the study groups to be compared using the GraphPad Prism 8.3.0c software (GraphPad Software Inc., La Jolla, USA). Statistical differences are designated as significant if the *p*-values are less than 0.05 (* *p* ≤ 0.05), and highly significant if the *p*-values are less than 0.01 (** *p* ≤ 0.01), less than 0.001 (*** *p* ≤ 0.001), or less than 0.0001 (**** *p* < 0.0001).

## 3. Results

### 3.1. Histopathological Analysis of the Cellular Migration

**Figure 2:**
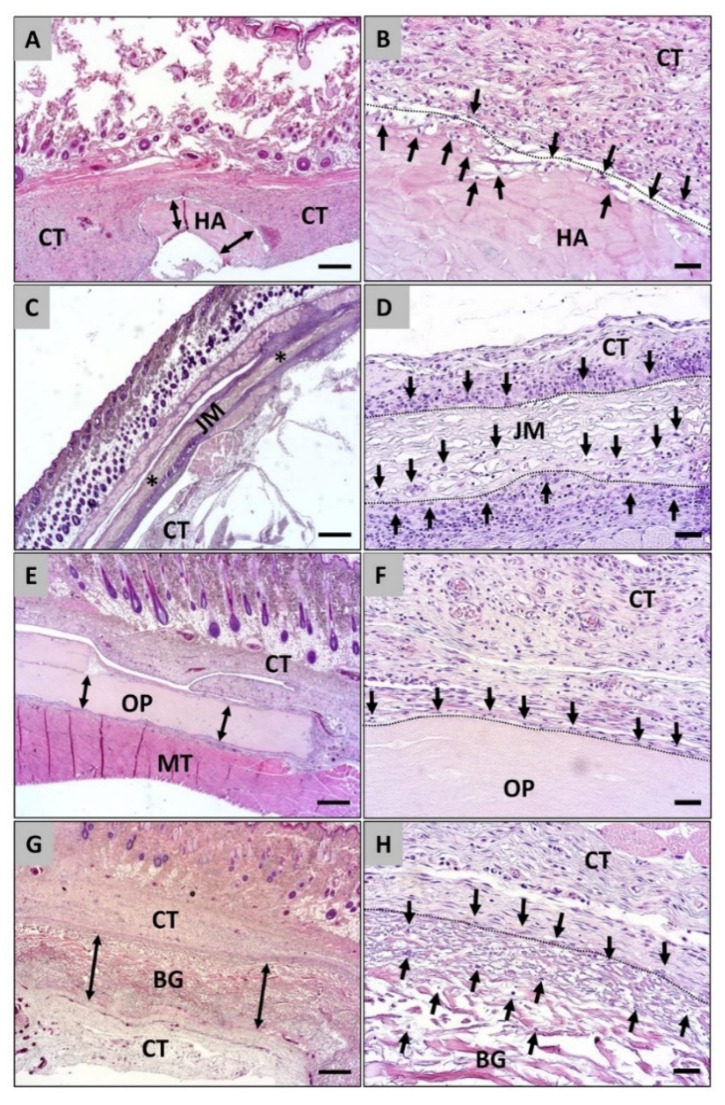
Histological images of the integration behavior and the tissue reactions of the four different collagen membranes implanted into the subcutaneous connective tissue (CT) at day 10 post implantationem. **Left** column: All four membranes (**A**,**E**,**G** = double arrows, **C** = asterisks) were detectable within their implantation beds without signs of fragmentation or material breakdown (H&E staining, 100× magnification, scalebar = 200 µm). **Right** column (**B**,**D**,**F**,**H**): Different cellular migration patterns (black arrows) were observed starting from the material surfaces (dashed lines). HA = hyaluronan/collagen membrane, JM = Jason^®^ membrane, OP = Ossix^®^ Plus membrane, BG = Bio-Gide^®^ membrane (H&E staining, 200× magnification, scalebar = 20 µm).

The histopathological analysis showed that all four membrane types were detectable within their subcutaneous implantation beds at day 10 post implantationem without signs of its fragmentation (Figure 2). The analysis furthermore revealed that the Coll-HA-membrane induced a mild inflammatory tissue reaction including only mononuclear cells, i.e., mainly macrophages beside lower numbers of granulocytes, lymphocytes, and fibroblasts (Figure 2A,B). Only single cells penetrated the membranes within the peripheral regions at this early study time point (Figure 2B). Also, in the study group of the Jason^®^ membrane a mild inflammatory tissue reaction involving only mononuclear cells, i.e., macrophages in concert with lymphocytes, granulocytes, and fibroblasts, were detected at this early time point (Figure 2C,D). Thereby macrophages and single lymphocytes have invaded the pores of the pericardium membrane up to the half of the membrane bodies (Figure 2,D). In the group of the OSSIX^®^ PLUS membrane a very mild inflammatory tissue reaction involving the same mononuclear cell types that did not penetrate the membrane bodies at day 10 post implantationem (Figure 2E,F). In the Bio-Gide^®^ group also a very mild inflammatory tissue response composed of mainly macrophages together with a few lymphocytes, granulocytes, and fibroblasts (Figure 2G,H). The analysis additionally showed that only single cells invaded this collagen membrane and most of the cells were located near to the material surfaces (Figure 2H).

**Figure 3:**
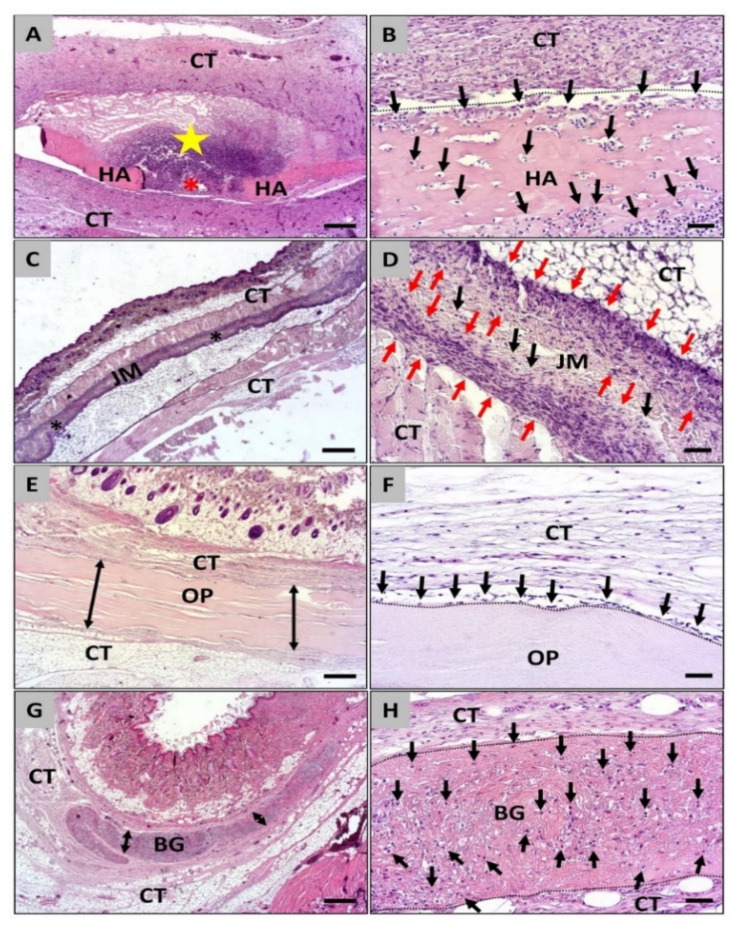
Histological images of the integration behavior and the tissue reactions of the four different collagen membranes implanted into the subcutaneous connective tissue (CT) at day 30 post implantationem. **Left** column: All four membranes (**A**,**E**,**G** = double arrows, **C** = asterisks) were detectable within their implantation beds and only the newly developed hyaluronan/collagen membrane (HA) showed signs of its fragmentation (red asterisk) associated with a granulation tissue with inflammatory cell infiltrate (yellow star). (H&E staining, 100× magnifications, scalebar = 200 µm). **Right** column (**B**,**D**,**F**,**H**): Also, at this time point varying cellular migration pattern (black) were observed starting from the material surfaces (dashed lines). HA = hyaluronan/collagen membrane, JM = Jason^®^ membrane, OP = Ossix^®^ Plus membrane, BG = Bio-Gide^®^ membrane, red arrows = cell wall within the collagen membrane (H&E staining, 200× magnification, scalebar = 20 µm).

The histopathologic analysis revealed that all four membrane types were observable within the subcutaneous connective tissue at day 30 post implantationem (Figure 3). At this time point the Coll-HA membrane induced a high extent of a material-related inflammatory tissue response in form of a granulation tissue (Figure 3A,B). In this material response, most often macrophages beside high numbers of (eosinophilic) granulocytes and lymphocytes as well as lower numbers of fibroblasts (Figure 3A,B). Thereby, the membranes showed most often signs of material fragmentations at this time point (Figure 3A). The analysis of the cellular migration of the membrane (parts) revealed that the materials were nearly completely invaded by macrophages (Figure 3B). In case of the Jason^®^ membrane still a mild inflammatory tissue reaction composed of only mononuclear cells, i.e., mainly macrophages beside single granulocytes, lymphocytes, and fibroblasts (Figure 3C,D). Higher numbers of mononuclear cells (mainly macrophages) migrated the membranes especially within the peripheral materials regions, while only single cells were migrated towards the membrane centers at this time point (Figure 3D). At day 30 post implantationem still a very mild inflammatory tissue reaction involving the single macrophages and fibroblasts were found in the group of the Ossix^®^ Plus membrane (Figure 3E,F). Thereby, no cells penetrated the membranes also at this study time point (Figure 3E,F). The analysis of the tissue reaction to the Bio-Gide^®^ membrane revealed still a very mild inflammatory tissue response including mainly macrophages together with single lymphocytes and fibroblasts (Figure 3G,H). Thereby, it was observable that single cells that could be assigned to the macrophage line have invaded the Bio-Gide^®^ membrane and were detectable distributed all over the membrane bodies (Figure 3H).

**Figure 4:**
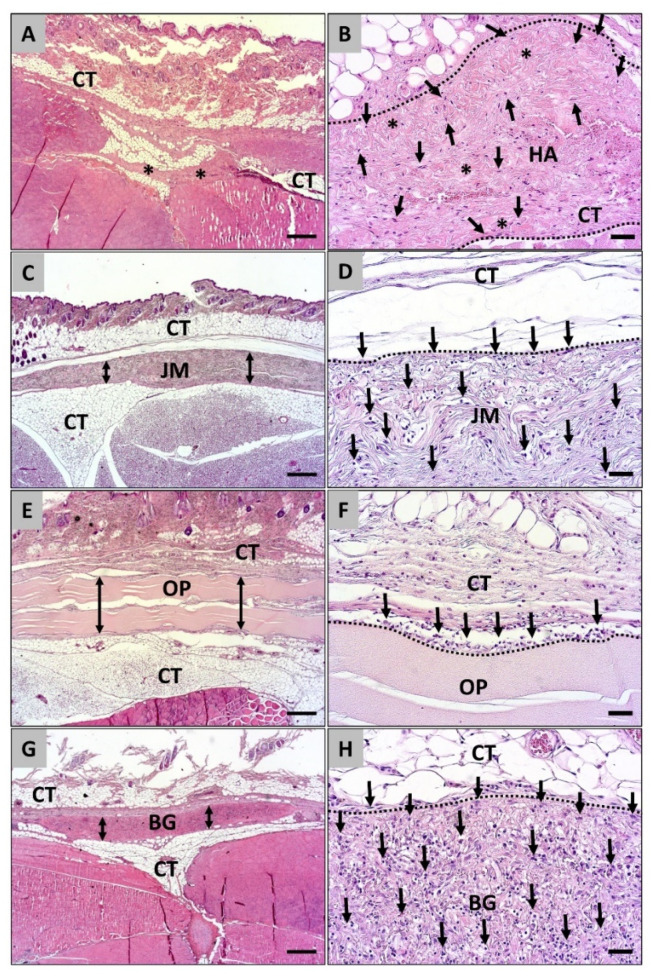
Histological images of the integration behavior and the tissue reactions of the four different collagen membranes implanted into the subcutaneous connective tissue (CT) at day 60 post implantationem. **Left** column: Only some remnants (asterisks) of the hyaluronan/collagen membrane (asterisks in **A**) were found at this late time point, while the other membranes (**C**,**E**,**G** = double arrows) were detectable within their implantation beds without signs of fragmentation or material breakdown (H&E staining, 100× magnification, scalebar = 200 µm). **Right** column (**B**,**D**,**F**,**H**): Different cellular migration patterns (black arrows) were observed starting from the material surfaces (dashed lines). HA = hyaluronan/collagen membrane, JM = Jason^®^ membrane, OP = Ossix^®^ Plus membrane, BG = Bio-Gide^®^ membrane (H&E staining, 200× magnification, scalebar = 20 µm).

At day 60 post implantationem the histopathologic analysis showed that only remnants of the Coll-HA-membrane were detectable within the subcutaneous tissue, while the other barrier membranes were still detectable without any signs of materials breakdowns or fragmentations (Figure 4). Thereby, the material remnants of the Coll-HA-membrane were detectable and seemed to be well integrated within the subcutaneous connective tissue and mononuclear cells (mainly macrophages) were distributed all over the material remnants (Figure 4A,B). Moreover, no further signs of a material-associated inflammatory tissue response were observable (Figure 4B). In the group of the Jason^®^ membrane only single mononuclear cells, i.e., mainly macrophages, were found at this study time point that were also loosely distributed over the complete material bodies (Figure 4C,D). Only minor histological signs of a material-related inflammatory tissue response could be detected (Figure 4D). In the Ossix^®^ Plus group a very mild tissue response including single macrophages and fibroblasts was observable related to the membranes and still no migration of cells into the materials was detected (Figure 4E,F). In the Bio-Gide^®^ group only minor histological signs of a material-associated inflammatory tissue reaction were observable including mainly macrophages and fibroblasts (Figure 4G,H). Furthermore, the analysis revealed that higher numbers of macrophages were findable distributed over the complete membrane bodies at this latest study time point (Figure 4H).

### 3.2. Histomorphometrical Analysis of the Cellular Migration

**Figure 5:**
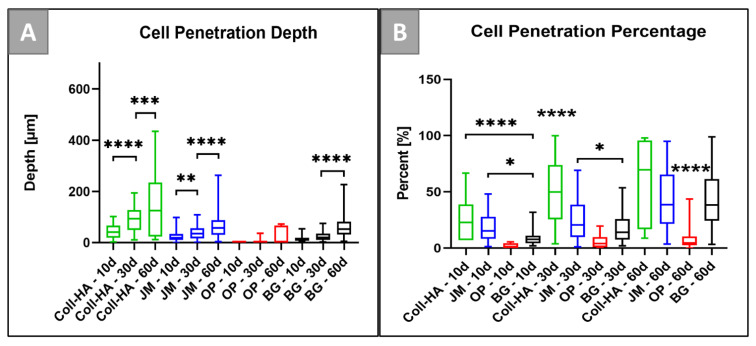
Histomorphometrical results of (**A**) the cellular penetration depth (in µm) and (**B**) the cellular penetration (in %) (* *p* < 0.05, ** *p* < 0.01, *** *p* < 0.001, and **** *p* < 0.0001).

Histomorphometrical analysis of the cellular penetration depth of the Coll-HA membrane exhibited an increasingly significantly higher cellular penetration between days 10 and 30 as well as between days 30 and 60 post implantationem (**** *p* < 0.0001 and *** *p* < 0.001, respectively) (Table 1 and Figure 5A). In case of Jason^®^ membrane, the cellular penetration depth increased significantly between day 10 and 30 as well as between day 30 and 60 post implantationem (** *p* < 0.01 and **** *p* < 0.0001, respectively) (Table 1 and Figure 5A). The Ossix^®^ Plus membrane exhibited very low cellular migration at all timepoints (Table 1). Thus, no significant differences were statistically recorded between the different time points (Figure 5A). Finally, BioGide^®^ membrane exhibited a highly significant increase of cellular penetration depth between the last two points (**** *p* < 0.0001) (Figure 5A).

The histomorphometrical analysis of the cellular penetration percentage in relation with the membrane thickness at the respective time points showed that the Coll-HA membrane underwent a significantly higher cellular penetration in comparison with the BioGide^®^ membrane at day 10 (**** *p* < 0.0001) (Table 1 and Figure 5B). Jason^®^ membrane also showed a less but still significantly higher cellular migration in comparison with the BioGide^®^ membrane at day 10 post implantationem (* *p* < 0.05) (Table 1 and Figure 5B). At day 30 post implantationem, the Coll-HA membrane showed a significantly higher cellular penetration in comparison to all other membranes (**** *p* < 0.0001). Furthermore, the Jason^®^ membrane displayed a higher cellular penetration compared to the BioGide^®^ membrane (* *p* < 0.05) (Figure 5B). At 60 days post implantationem, the Coll-HA membrane, the Jason^®^ membrane, and the BioGide^®^ membrane showed elevated cellular penetration percentages and all values were significantly higher than in the Ossix^®^ Plus group (**** *p* < 0.0001) (Table 1 and Figure 5B).

## 4. Discussion

A novel histomorphometrical approach was evaluated to analyze the cellular invasion of collagen-based barrier membranes. This topic is still of high importance as cellular invasion is mainly correlating with the barrier functionality of a resorbable collagen-based membranes, whose main functionality is the separation of hard and soft tissue compartments in the context of guided bone regeneration (GBR) [35]. In the context of larger defects, resorbable membranes for GBR require a longer barrier functionality and standing time. Thus, materials research is constantly searching for methods (such as various cross-linking techniques) to extend the service life of bioresorbable GBR membranes while maintaining a good tissue compatibility [5]. A broad variety of mostly preclinical studies have been conducted to analyze the cellular migration pattern of barrier membranes both in vitro and in vivo [6,20,36,37]. However, only qualitative data regarding this topic are normally reported, as no quantitative methodology to measure the cell migration behavior has been reported until now [6,20,36,37].

In order to answer this open question of quantitative analytics, especially with regard to this problem, a novel histomorphometrical analysis method was developed and tested. This methodology was tested on three commercially available collagen-based barrier membranes and one newly developed collagen membrane that was noncovalently combined with hyaluronic acid.

Initially, the histopathological analysis revealed that all four membranes were intact at 10 days post implantationem. Thereby, in the groups of the Coll-HA membrane and the BioGide^®^ membrane only a peripheral cell invasion was noted, while in case of the Jason^®^ membrane also a migration towards the centers of the membranes was observable. In contrast, no cellular migration in the group of the Ossix^®^ Plus membrane group was notable at this early time point. At day 30 post implantationem the Coll-HA membrane started to fragment and only remnants of the membrane were observable. Thereby, the membrane, and even its remnants, allowed a high invasion of cells. Both the Jason^®^ membrane and the BioGide^®^ membrane allowed for a mainly peripheral cellular migration that most often also reached the central material parts. Only in case of the Ossix^®^ Plus membrane group no cellular migration was detectable. At day 60 days post implantationem only material remnants that were fully invaded by cells were found in the Coll-HA membrane group. Also, in the groups of the Jason^®^ membrane and the BioGide^®^ membrane an increased cellular migration took place, while only a slight peripheral cell migration was still found in the Ossix^®^ Plus membrane group.

Histomorphometrically, both the Coll-HA membrane and the Jason^®^ membrane exhibited an increasing cellular penetration depth within the time course of the study. Comparatively with BioGide^®^, the Jason^®^ membrane exhibited a high infiltration only at the later study time points. This observation can be explained by the bilayer nature of BioGide^®^ and that one layer is hindering the penetration of the second. Radenkovic et al. observed that BioGide^®^ has two layers, one of which degrades faster than the other, meaning that its degradation rate is not linear and drops after the less dense layer is absorbed at day 30 post implantationem [6]. Interestingly, the data of the present study underline this hypothesis as the cellular penetration depth increases significantly after this timepoint.

To be able to statistically compare the different cellular penetration degrees of the four membranes, percentages of the respective thicknesses were calculated. This step allows for the normalization of the membrane thicknesses as these membranes have reported different thickness baselines [6,34]. This calculation showed that the Coll-HA membrane exhibited a significantly higher cellular invasion starting from the earliest timepoint, even before the fragmentation has started. In contrast, the Ossix^®^ Plus membrane displayed the lowest cellular penetration degree and at day 60, the difference between Ossix^®^ Plus and the other membranes was significantly higher.

Finally, the native collagen membranes (Jason^®^ membrane and BioGide^®^ membrane) displayed similar percentages; however, Jason^®^ had a higher penetration of cells at the first timepoints. Nevertheless, the percentages were comparable for both membranes at the last timepoint. Altogether, these histomorphometrical observations completely support the histopathological analyses of these membranes but in a quantitative manner. Further studies have to prove this methodology for its reproducibility in vivo—but also for its suitability in vitro studies. In this context, the migration of cell types such as fibroblasts, which are “model cells”, are investigated in the context of cytocompatibility studies that are required for the approval of medical devices based on DIN ISO 10993-5/-12, which is also part of the medical device regulation (MDR) [38]. Moreover, a study should prove the comparative suitability of this methodology in in vitro and in vivo studies with regard to “animal-friendly studies” in accordance with the 3Rs principle [39].

Finally, the question of the induction factors of the different integration pattern of the analyzed membranes remains. In the case of the two native collagen membranes Jason^®^ membrane and BioGide^®^, the presented results led to the conclusion that these commercially available native collagen membranes cause less severe material-induced inflammation and are therefore absorbed slower and retain their barrier functionality for a longer period while integrating with the host’s tissue. These observations are fully in line with previous preclinical study results that show the tissue integration of both membranes based on physiological processes involving cellular elements of the collagen metabolism, i.e., fibroblasts, macrophages, and eosinophils [6,18,20,37,40]. Thereby, these membranes “behave” similarly to bone substitute materials following the principle of “creeping substitution” as the biomaterials are gradually replaced by connective tissue while maintaining their barrier functionality. Moreover, a broad variety of clinical studies support these observations as both membranes have proven to be barrier materials leading to satisfying clinical results during Guided Bone Regeneration (GBR) applications [15,16,17,18,19,20].

Furthermore, the results, combining the histopathological observations with the histomorphometrical evaluation, the extended resorption time of the Ossix^®^ Plus membrane due to the crosslinking by means of ribose can be confirmed in this study, which is in concert with previous literature [6,34]. The special cross-linking process based on glycation, which is a natural reaction of collagen fibers during aging [41]. This reaction pattern is supposed to be an optimal basis for sugar crosslinking of collagen, resulting in a barrier membrane that may provide a sufficiently long barrier functionality that does not provoke exaggerated inflammatory tissue reactions within the peri-implant tissue.

In this context, a recently published study by Radenkovic and colleagues reported that this sugar-crosslinked membrane lacked in cellular infiltration and provides a very slow degradation pattern combined with a suitable biocompatibility and stability up to 60 days post-implantation [6]. Thus, it was concluded that this membrane might be suitable for application in GBR as a biomaterial with exclusive barrier functionality, similar to non-resorbable options [7]. Additionally, Rothamel et al. analyzed crosslinked barrier membranes subcutaneously including the Ossix^®^ Plus membrane, and observed similar points in terms of cellular invasion and intactness of Ossix^®^ Plus [34]. Finally, the reported data substantiate the previous study results won via histological observation.

The addition of hyaluronic acid, that was used in the newly developed membrane, cannot compete with the results of the Ossix^®^ Plus membrane (and also not with the native membranes) as it shows deficiencies particularly in the area of stability. This was comparatively shown in the present study by the histological observations and quantitatively using the newly developed histomorphometrical approach. This observation can lead to a hypothesis that hyaluronic acid in this case is a pro-inflammatory mediator that triggered the increased migration and phagocytosis, causing premature fragmentation of the membrane. For further insight, the polarization of the residing macrophages that could provide the level of phagocytosis activity should be analyzed. However, high molecular weight HA has been reported to trigger anti-inflammatory, and non-phagocytosing, macrophage polarization in vitro [42]. Furthermore, Pröhl et al. also investigated in vivo high molecular weight HA that was combined with a xenogeneic bone graft and their results suggest that the HA was degraded upon implantation and prior to it having any molecular influence [26]. Moreover, a study conducted by Sieger et al. showed that that only the addition of high doses of HY to a biphasic bone substitute significantly decreases the occurrence of pro-inflammatory macrophages [27]. Therefore, the literature dismisses the hypothesis that the HA addition to the collagen might lead to a transition to pro-inflammatory mediation. Another explanation could be that the Coll-HA membrane did not have enough stability and was rapidly broken down enzymatically. With the assumption that the hyaluronic acid was immediately released from the biomaterial and resorbed, without having any influence on the host tissue reaction. This breakdown then allowed for higher cellular infiltration. Altogether, these results underline that the Coll-HA membrane can only be used to a limited extent for dental use as part of a GBR procedure, as it does not achieve the minimum resorption time of around 3–4 months required for many indications and provokes a higher inflammatory cellular and tissue response [5]. The use of this membrane could thus lead to a premature exposure of the defect area to soft tissue, which in turn can end up in less osteogenesis [16]. Non-osteogenic cells of the surrounding tissue would have access to the defect area too quickly and would therefore not give the osteogenic cells the time they need to regenerate. Soft tissue would grow in before the defect is filled with the required amount of new bone. A failure of the attempted bone augmentation would have to be expected with a high degree of probability under these conditions.

On the other hand, short-lived resorbable membranes can be used in the field of periodontal surgery as well as soft tissue regeneration. For example, it can be used to recover gingival recession (as a substitute to autografts like free gingival grafts or connective tissue grafts) [43] or for minor alveolar defects such as extraction [44]—and thus in cases that require a fast tissue ingrowth. The perforation of the Schneiderian membrane during sinus lifts is very common and can be managed with resorbable membranes that have short standing-time since the Schneiderian membrane is usually healed in 4 weeks [45]. Other applications might be also conceivable. Postoperative intraperitoneal adhesion prophylaxis could be a possible area of application for the Coll-HA membrane. This type of application would be particularly of interest after surgery on the abdomen and the abdominal wall. In this process, an abnormal connection between the intraperitoneal structures could possibly be prevented by the formation of fibrous strands [46].

Limitations of the present study could be found in the fact that only single slides from every implantation bed were analyzed. Thus, this approach limits the informative value to some extent, so that the analysis of several sectional preparations per implant would appear to be most useful. However, such an approach—especially in the case of manual evaluation—would mean considerable additional expense, both financially and timely. A way around this problem would therefore be the usage of (semi-) automated scripts for software suites such as ImageJ, in order to minimize the evaluation time. Another possibility would be to use other 3D-based imaging techniques for hard and the soft tissues, such as Computerized Tomography (CT), Cone Beam Computerized Tomography (CBCT), Micro Computerized Tomography (MCT), to obtain the cellular migration over the complete material area. In summary, the results of the present study have shown that the applied addition of HA does not lead to a prolonged standing and resorption time. This result was elaborated qualitatively through histopathological analysis and further proved quantitatively through the newly developed histomorphometrical approach to measure the cellular infiltration. The results showed that the membrane was almost completely resorbed by day 60 post implantationem. Moreover, the novel preclinical comparison of the integration patterns between various resorbable collagen membrane types were accomplished by a novel histomorphometrical approach that allowed the quantification of histopathological observations. Via this approach, it was possible to semi-quantify the different levels of cellular penetration of GBR membranes that were only qualitatively analyzed through histopathological approaches before.

The results of the study furthermore revealed that the addition of hyaluronic acid addition to collagen does not lead to a prolonged standing time, but an increased integration of a collagen-based biomaterial. Therefore, it can only partially be used in the dental field for indications that require a fast and guided cell or tissue influx such as periodontal regeneration processes.

## Figures and Tables

**Table 1:** Results of the cellular penetration in the four different study groups (mean values ± standard deviation).

	Coll-HA Membrane		Jason^®^ Membrane		Ossix^®^ Plus Membrane		BioGide^®^ Membrane	
	µm	%	µm	%	µm	%	µm	%
10 days	42.7 ± 27.6	26.2 ± 18.6	26.0 ± 21.9	18.5 ± 11.9	0	0	13.6 ± 8.3	8.0 ± 4.8
30 days	92.4 ± 48.9	50.8 ± 28.1	38.2 ± 25.5	25.2 ± 17.9	11.9 ± 20.8	5.8 ± 6.6	25.3 ± 17.8	18.4 ± 12.9
60 days	138.5 ± 130.2	58.26 ± 38.2	68.2 ± 54.3	42.6 ± 24.8	26.5 ± 36.5	8.9 ± 12.3	60.2 ± 42.1	41.6 ± 24.8

## Data Availability

The data presented in this study are available in article.
